# Translesion Synthesis in Plants: Ultraviolet Resistance and Beyond

**DOI:** 10.3389/fpls.2019.01208

**Published:** 2019-10-09

**Authors:** Ayako N. Sakamoto

**Affiliations:** Department of Radiation-Applied Biology Research, National Institutes for Quantum and Radiological Science and Technology, Takasaki, Japan

**Keywords:** translesion synthesis, UV, mutation, DNA damage, genome stability

## Abstract

Plant genomes sustain various forms of DNA damage that stall replication forks. Translesion synthesis (TLS) is one of the pathways to overcome stalled replication in which specific polymerases (TLS polymerase) perform bypass synthesis across DNA damage. This article gives a brief overview of plant TLS polymerases. In *Arabidopsis*, DNA polymerase (Pol) ζ, η, κ, θ, and λ and Reversionless1 (Rev1) are shown to be involved in the TLS. For example, AtPolη bypasses ultraviolet (UV)-induced cyclobutane pyrimidine dimers *in vitro*. Disruption of AtPolζ or AtPolη increases root stem cell death after UV irradiation. These results suggest that AtPolζ and ATPolη bypass UV-induced damage, prevent replication arrest, and allow damaged cells to survive and grow. In general, TLS polymerases have low fidelity and often induce mutations. Accordingly, disruption of AtPolζ or AtRev1 reduces somatic mutation frequency, whereas disruption of AtPolη elevates it, suggesting that plants have both mutagenic and less mutagenic TLS activities. The stalled replication fork can be resolved by a strand switch pathway involving a DNA helicase Rad5. Disruption of both AtPolζ and AtRAD5a shows synergistic or additive effects in the sensitivity to DNA-damaging agents. Moreover, AtPolζ or AtRev1 disruption elevates homologous recombination frequencies in somatic tissues. These results suggest that the Rad5-dependent pathway and TLS are parallel. Plants grown in the presence of heat shock protein 90 (HSP90) inhibitor showed lower mutation frequencies, suggesting that HSP90 regulates mutagenic TLS in plants. Hypersensitivities of TLS-deficient plants to γ-ray and/or crosslink damage suggest that plant TLS polymerases have multiple roles, as reported in other organisms.

## Introduction

**Figure 1:**
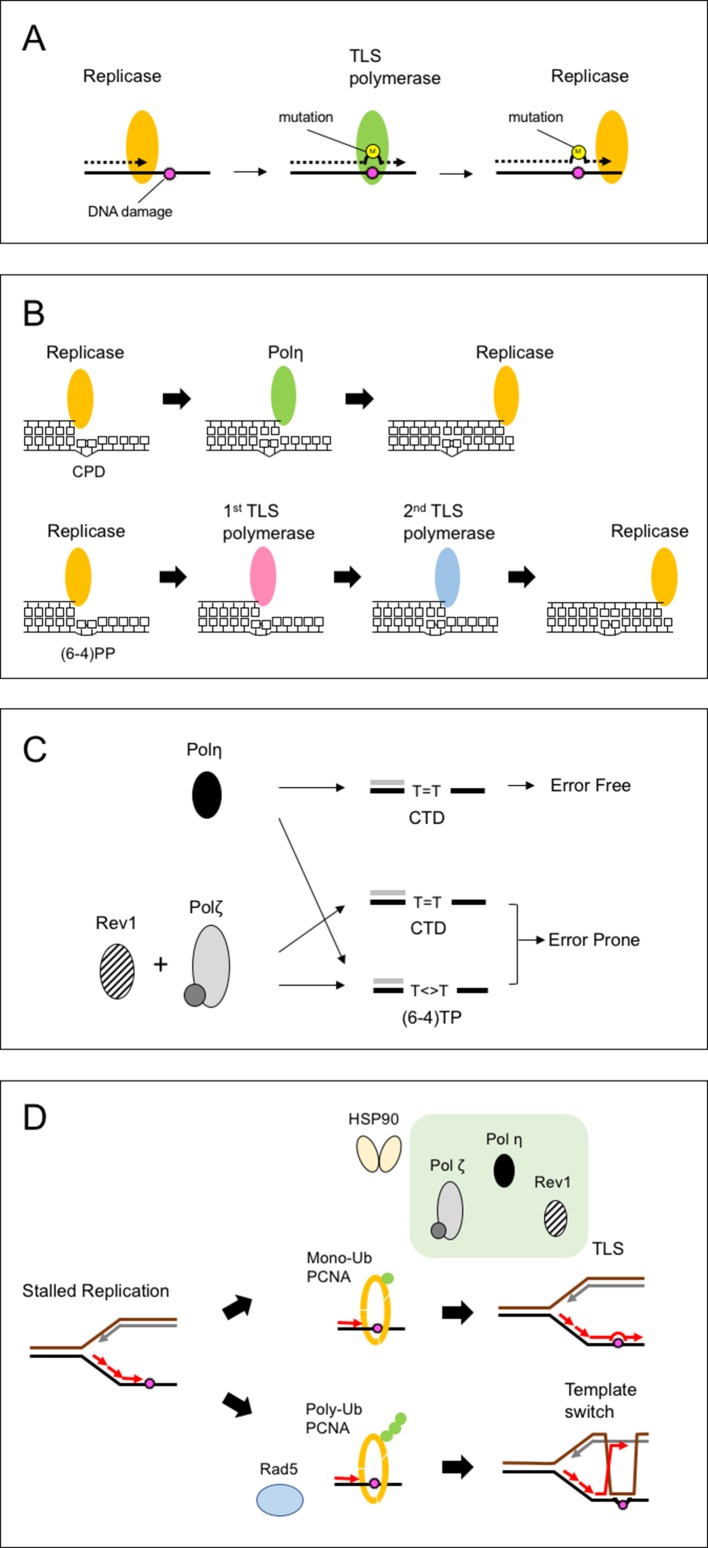
Schematic of translesion synthesis (TLS). **(A)** Concept of TLS. When encountering DNA damage, the replicase stalls before the damage. TLS polymerase replaces the replicase and inserts one or more nucleotides opposite the damage. Because of the low fidelity, TLS polymerase incorporates one or more incorrect nucleotides, resulting in base substitutions, frameshifts, or other types of mutation. **(B)** Proposed model for the bypass of two major forms of ultraviolet (UV) damage. The model was proposed from the biochemical activities of TLS polymerases. The cyclobutane pyrimidine dimer (CPD) is efficiently bypassed by Polη (upper). However, no polymerase can complete the bypass of (6-4) photoproducts [(6-4)PP] by itself. Thus, (6-4)PPs may be bypassed by two polymerases, incorporating nucleotides one after the other (lower). **(C)** A model for UV-induced mutagenesis at the TT site in plants. The cyclobutane TT dimer (CTD) is efficiently bypassed by Polη in an error-free manner; any misincorporation is removed by replicases. In contrast, Polζ and Rev1 are involved in the error-prone bypass for both CTD and (6-4) TT photoproducts [(6-4)TP]. Polη cannot complete the bypass of (6-4)TP, so error-prone bypass is achieved by Polζ. **(D)** A model for damage tolerance mechanism in plants. The stalled replication fork is processed by either of two pathways: mutagenic synthesis by specific TLS polymerases or accurate synthesis using an intact template (template switch). The stalled replication fork signals the modification of PCNA. When PCNA is monoubiquitinated, the TLS polymerases interact with the Ub-PCNA and are recruited to the replication fork. The stalled replication fork also signals the transfer of Polη and Rev1. The 90-kDa heat shock protein (HSP90) promotes TLS activity through interaction with TLS polymerases. When TLS is deficient or reduced by depletion of HSP90, Rad5-dependent polyubiquitination of PCNA leads to a template switch, which causes genome instability.

Accurate replication of genomic DNA is vital for maintaining genome integrity. However, genomic DNA sustains various forms of damage caused by internal and external agents. Ultraviolet (UV) light is a major cause of DNA damage for land plants. It induces the formation of covalent bonds between the two adjacent pyrimidines. The two major products of UV damage, cyclobutane pyrimidine dimers (CPDs) and pyrimidine (6-4) pyrimidone photoproducts [(6-4)PPs], are quickly repaired by the action of CPD and 6-4 photolyases in plant cells (Britt, 1999; Li et al., 2010; Hitomi et al., 2012). In addition, nucleotide excision repair (NER) plays an important role in removing UV damage (Kimura et al., 2004; Kunz et al., 2005; Canturk et al., 2016). Nevertheless, the remaining damage is toxic for cells because it distorts the template structure and prevents replication. This stalled replication creates a fragile single-strand region that easily leads to double-strand breaks (DSBs), so organisms have multiple pathways to solve the stalled replication fork. Translesion synthesis (TLS) is one such pathway in which specific polymerases (TLS polymerase) are recruited to the replication machinery and perform the bypass synthesis across the DNA damage (Vaisman and Woodgate, 2017). **Figure 1A** illustrates the concept of TLS activity. When encountering DNA damage, the replicative polymerase (replicase) stalls because of distorted helix geometry. TLS polymerase, carrying a flexible active site, replaces the replicase and inserts one or more nucleotide(s) opposite the damage. Because of the relaxed constraints of these active sites, the TLS polymerase has a low fidelity and often incorporates one or more incorrect nucleotide(s) that can be removed by the exonuclease activity of replicases or the mismatch repair mechanism. However, unremoved errors result in base substitutions, frameshifts, or other types of mutation. This mutagenic nature of TLS has been linked to the senescence, carcinogenesis, and evolution of organisms.

It is more than a decade since the first report of TLS in plants. The accumulation of reports from multiple groups has clarified the roles and importance of TLS not only in UV resistance but also in the maintenance of genome stability in plants. This mini-review aims to summarize 1) TLS activity in plants in comparison with that in other organisms, 2) the contribution of TLS activity to plant responses to DNA-damaging stresses, and 3) possible other functions of TLS polymerases, which may unveil novel damage-resistant mechanisms in plants.

## DNA Polymerase Family Members in Plants

DNA polymerases are classified into seven families based on their amino acid sequence similarity (Ishino and Ishino, 2014). Eukaryotes have Family A, B, X, and Y polymerases, whereas Family C polymerases are only seen in bacteria and Family D and E polymerases only in archaea. *Arabidopsis* has at least 11 polymerases classified into five families based on comparisons with human and yeast homologs (**Table 1**). The representative member of Family A polymerases is *Escherichia coli* polymerase I, which was the first DNA polymerase to be identified (Kornberg et al., 1956). Eukaryotic members of this group are polymerase γ (Polγ) and DNA polymerase θ (Polθ). *Arabidopsis* also has homologs of two prokaryotic-type DNA polymerases, PolI-like A and B (Parent et al., 2011), as well as AtPolθ, which was originally isolated as the causative gene of the short-root mutant *tebichi* (Inagaki et al., 2006). Family B polymerases include *E. coli* Pol II and eukaryotic polymerases α (Polα), δ (Polδ), and ε (Polε), which are involved in the replication of nuclear DNA. Polα, δ, and ε are conserved in *Arabidopsis* (Ronceret et al., 2005; Shultz et al., 2007; Liu et al., 2010; Iglesias et al., 2015; Pedroza-Garcia et al., 2016). This family includes DNA polymerase ζ, the first identified TLS polymerase that is also conserved in *Arabidopsis* (Sakamoto et al., 2003). Family X is only conserved in eukaryotes: its representative polymerase is Polymerase β, which is involved in base excision repair. Humans have four members in Family X (Polβ, Polλ, Polμ, and terminal deoxytransferase), whereas plants only have Polλ, which is phylogenetically distant from the Polλ of other organisms (Filée et al., 2002; Pavlov et al., 2006). Family Y carries the largest number of TLS polymerases, including *E. coli* Pol IV and V; eukaryotic Polη, Polκ, Polι; and Rev1 (Ohmori et al., 2001). Rev1 was originally isolated as a responsible gene for yeast *reversionless1* mutant, which carries a deoxycytidyl transferase activity (Nelson et al., 1996). Homologs of Polη, Polκ, and Rev1 are found in *Arabidopsis* (Takahashi et al., 2007).

**Table 1:** DNA polymerases in *Arabidopsis*^a,b^.

Family	Category	Subunit	*A. thaliana* Gene ID	Reference	Function
**A**	DNA polymerase IA DNA polymerase IB	POLIA POLIB	At3g20540 At1g50840	Parent et al., 2011	Replication of organellar DNA, TLS Replication of organellar DNA, TLS
	DNA polymerase θ	POLQ	At4g32700	Inagaki et al., 2006	Repair of crosslink damage DSB repair TLS
**B**	DNA polymerase α	POLA1 POLA2 POLA3 POLA4	At5g67100 At1g67630 At1g67320 At5g41880	Shultz et al., 2007; Liu et al., 2010	Replication
	DNA polymerase δ	POLD1 POLD2 POLD3 POLD4	At5g63960 At2g42120 At1g78650 At1g09815	Shultz et al., 2007; Iglesias et al., 2015	Replication
	DNA polymerase ε	POLE1 POLE2 POLE3 POLE4	At1g08260 At2g27120 At5g22110 At1g07980 At5g43250 At2g27470	Ronceret et al., 2005; Pedroza-Garcia et al., 2016	Replication
	DNA polymerase ζ	REV3 REV7	At1g67500 At1g16590	Sakamoto et al., 2003; Takahashi et al., 2005	TLS, Repair of crosslink damage DSB repair
**X**	DNA polymerase λ	POLL	At1g10520	Uchiyama et al., 2004	Repair synthesis TLS
**Y**	DNA polymerase η	POLH	At5g44740	Santiago et al., 2006	TLS, Repair of crosslink damage
	DNA polymerase κ	POLK	At1g49980	García-Ortiz et al., 2004	TLS
	Rev1	REV1	At5g44750	Takahashi et al., 2005	TLS, Repair of crosslink damage

^a^Homologs for DNA polymerase σ are not listed here because opinions are divided whether Polσ has a DNA polymerase activity or not. ^b^Organellar DNA primases are not listed here.

Most recently, it has been shown that some members of the Archea-Eucaryotic Primase superfamily, such as human PrimPol, perform bypass synthesis across DNA damage (Iyer et al., 2005; Bianchi et al., 2013; Guilliam et al., 2015). *Arabidopsis* has a herpes-pox type primase (Iyer et al., 2005), although its function has not yet been investigated.

## Isolation of Translesion Synthesis Polymerases Based on Ultraviolet Resistance

A UVB-sensitive mutant *rev3* was isolated in *Arabidopsis* by screening ion-beam mutagenized seedlings under non-photoreactivating conditions (Sakamoto et al., 2003). The responsible gene, *AtREV3*, encodes a homolog of the catalytic subunit of DNA polymerase ζ (Polζ). DNA replication in the *rev3* root meristem was reduced after UVB irradiation (Sakamoto et al., 2003). *AtREV7* and *AtREV1* encode a regulatory subunit of Polζ and a Family Y polymerase, respectively (Takahashi et al., 2005). The *rev7* and *rev1* plants showed reduced growth compared with wild-type plants under chronic UVB irradiation (Takahashi et al., 2005). *AtPOLH* encodes a homolog of DNA polymerase η (Polη) that complements the yeast *rad30* mutant (Santiago et al., 2006). Disruption of Polζ and Polη had an additive effect on *Arabidopsis* root growth after UVB treatment (Anderson et al., 2008). Moreover, cell death was induced at root stem cells, and the number of mitotic cells was reduced severely in the UV-irradiated Polζ- and Polη-deficient plants (Curtis and Hays, 2007). This series of studies showed that these polymerases are important in plant UV resistance. The polymerases allow DNA replication to continue, saving the stem cell from cell death and maintaining growth in the presence of harmful UV irradiation.

## Damage Bypass Activities of Translesion Synthesis Polymerases

TLS activity has been investigated *in vitro* using purified or recombinant polymerases and synthetic damage-inducing templates, such as cyclobutane TT dimer (CTD) and (6-4)TT photoproducts [(6-4)TP]. These analyses revealed that the bypass efficiency is dependent on both the type of damage and the polymerases involved (**Figure 1B**). For example, yeast and human Polη bypasses CTD efficiently (Johnson et al., 1999; Masutani et al., 1999), but Polη only inefficiently bypasses (6-4)TP (Johnson et al., 2001). In humans, DNA polymerase ι (Polι) inserts a nucleotide opposite 3′-T in the (6-4)TP (Vaisman et al., 2003). The 3′-end is thought to be elongated by the second polymerase (Polζ, Polκ, or Polθ), which has 3′-end elongation activity (Prakash et al., 2005, Seki and Wood, 2008). The subsequent *in vivo* analyses suggest that the UV damage at CC or CT sequence are also bypassed by a similar one- or two-step mechanism. Thus, TLS involves the multiple switching of polymerases at the replication site (**Figure 1B**; Prakash and Prakash, 2002; Bebenek and Kunkel, 2004).

The bypass activity of AtPolη for the major UV damage was examined by two groups who showed that AtPolη bypasses the CTD *in vitro* (Anderson et al., 2008; Hoffman et al., 2008). The activity of AtPolη is comparable to that of human Polη when examined at optimum salt concentration and temperature, and HsPolη, ScPolη, and AtPolη do not bypass (6-4)TP (Hoffman et al., 2008). Research has also been done on other types of DNA damage: AtPolκ inserted an A/C opposite 8-oxoG, a common form of oxidative damage induced by reactive oxygen species (García-Ortiz et al., 2007). Deletion of the C-terminal domain elevates the processivity and fidelity of AtPolκ, suggesting that the C-terminal domain regulates the activities of this polymerase through interactions with other proteins (García-Ortiz et al., 2004; García-Ortiz et al., 2007). DNA polymerase λ bypassed 8-oxoG in both error-free (dC insertion) and error-prone (dA insertion) manners (Amoroso et al., 2011). AtRev1 inserted a C opposite an apurin/apyrimidine (AP) site (Takahashi et al., 2007), which is formed by spontaneous depurination or occurs as an intermediate in the base excision repair process (Boiteux and Guillet, 2004). AtPolIA and AtPolIB have also been shown to bypass the AP site *in vitro* (Baruch-Torres and Brieba, 2017).

## Detection of Mutations Induced by Translesion Synthesis

Mutations induced by TLS have been investigated in *in vivo* assay systems (Lawrence and Christensen, 1978; Lawrence and Christensen, 1979; Roche et al., 1994; Harfe and Jinks-Robertson, 2000; Yu et al., 2001; Bresson and Fuchs, 2002; Kozmin et al., 2003; Gibbs et al., 2005; Szüts et al., 2008; Yoon et al., 2009; Yoon et al., 2010). In yeast, the deletion of Polζ or Rev1 reduces the UV-induced mutation frequency (Lawrence and Christensen, 1978, Lawrence and Christensen, 1979), whereas the deletion of Polη increases the frequency (Yu et al., 2001; Kozmin et al., 2003). These observations are not consistent with the *in vitro* characteristics of Polζ and Polη because Polη is less accurate than Polζ when replicating undamaged DNA (McCulloch et al., 2007; Zhong et al., 2006). Comprehensive analysis of *in vitro and in vivo* data suggested that Polη bypasses CTD with some errors, which are removed by the exonuclease activity of other polymerase(s) (McCulloch and Kunkel, 2008). Prakash et al. (2005) suggest that yeast Polη bypasses CPD at CC or CT sequence in an error-free manner. However, Pol η seems to induce C to T transition by inserting dA opposite deaminated C or mC in CPD (Ikehata and Ono, 2011). Yeast and mammalian Polη bypass (6-4)TP in an error-prone manner (Bresson and Fuchs, 2002; Yoon et al., 2010). It is suggested that Polζ contributes to the mutagenic bypass of (6-4)PP by extending the mismatched primer end caused by the action of Polη or other polymerases (Prakash et al., 2005; Hirota et al., 2010). Thus, the mutation frequency depends on the polymerases available, damage type, sequence context, and the assay system, and so on.

In plants, the reversion frequencies in *Arabidopsis* plants were measured using β-glucuronidase (GUS)-based markers (Kovalchuk et al., 2000; Nakagawa et al., 2011a; Nakagawa et al., 2011b).

The markers carry a G-T mutation, which corresponds to the 3’-T of TT sequence, a possible target of UV dimer. A misincorporation of dC opposite 3’-T leads to detect a reversion (a T to G transversion). When irradiated with UVB, the Polζ- and Rev1-deficient plants made fewer reversions in somatic cells compared with wild-type plants. By contrast, the Polη-deficient plant showed higher reversion frequencies than wild-type plants, which were reduced in Polζ and Polη double-deficient plants. From these results, the authors proposed a model in which *Arabidopsis* has two TLS pathways for responding to UV damage: a more mutagenic pathway involving Polζ and Rev1 and a less mutagenic pathway involving Polη (Nakagawa et al., 2011a). Polη bypasses CTD in an error-free manner (**Figure 1C**). Polζ and Rev1 bypass both CTD and (6-4)TP in an error-prone manner. The Polη inserts a nucleotide opposite (6-4)TP, which is extended by Polζ and causes the mutation. Since the bypass activity across (6-4)TP is low anyway, the minor dC insertions would be detected in this assay system. However, other explanations are possible, for example, when UV induces a double-strand break near the TT sequence, which is wrongly repaired and causes a mutation. Also, further analysis by employing a C-containing marker is necessary to profile UV-induced mutations in plants.

## Regulation of Translesion Synthesis

Maintenance of the replication fork is crucial because stalled replication forks easily lead to strand breaks. It has been suggested that a stalled replication fork signals the modification of proliferating cell nuclear antigen (PCNA), which triggers the switching of replicase to TLS polymerase (Stelter and Ulrich, 2003; Kanao and Masutani, 2017). That is, when the PCNA is monoubiquitinated, the replicase detaches from PCNA and TLS polymerases are recruited to the replication site to perform the bypass of damaged DNA, whereas polyubiquitinated PCNA leads to the strand switch pathway. The mammalian Polη, Polκ, and Rev1 have been shown to interact with monoubiquitinated PCNA through the UBZ or UBM motif located in the C-terminal (Bienko et al., 2005; Wood et al., 2007). Moreover, Rev1 has also been shown to interact with other TLS polymerases (Guo et al., 2003) and is suggested to function as a bridge through which the best polymerase for TLS is selected (Boehm et al., 2016).

*Arabidopsis* has two copies of PCNA, but only AtPCNA2 complements the yeast *pol30* mutant (Anderson et al., 2008). The AtPolη has a UBM motif and two PIP repeats but does not have a UBZ motif conserved in animal and yeast Polηs. The mutant AtPolη disrupted in PIP1, PIP2, or UBM still interacts with *Arabidopsis* PCNA2 but does not fully complement yeast *rad30* cells (Anderson et al., 2008). Both *Arabidopsis* PCNAs interact with ubiquitin in *N. benthamiana* cells and are ubiquitinated *in vitro* (Strzalka et al., 2013). The AtREV1 interacts with PCNA2, AtPolη, and AtREV7, a regulatory subunit of AtPolζ in yeast (Sakamoto et al., 2018). The processivity of rice Polλ is stimulated in the presence of PCNA (Uchiyama et al., 2004). Moreover, when *Arabidopsis* Polλ bypasses 8-oxoG, the ratio of error-free (dC insertion) to error-prone (dA insertion) bypass changed depending on its interaction with PCNA2 (Amoroso et al., 2011). These results suggest that the modification of PCNA leads to the switching from replicase to the appropriate TLS polymerase in plants.

It has been suggested that stalled replication in plants is also resolved by a Rad5-dependent strand switch pathway (Wang et al., 2011). The *rev3* and *rad5a* mutations caused synergistic or additive effects on root growth in plants exposed to UV, MMS, or crosslink agents compared with plants containing each single mutation (Wang et al., 2011). The *rad5a* plant failed to induce homologous recombination events after bleomycin treatment (Chen et al., 2008). By contrast, *rev3* and *rev1* plants induced significantly more recombination events after UV irradiation (Sakamoto et al., 2018). If AtRAD5a and AtREV3 work *via* two alternative pathways, the elevation of recombination activities in *rev3* and *rev1* plant could be due to the activation of a RAD5-dependent pathway (**Figure 1D**).

## Translesion Synthesis and Heat Shock Protein 90

The 90-kDa heat shock protein (HSP90) is an evolutionarily conserved molecular chaperone that stabilizes and activates various proteins involved in homeostasis, transcriptional regulation, chromatin remodeling, and DNA repair (Pennisi et al., 2015). The *Arabidopsis* genome has four copies of cytosolic HSP90 and three copies of organellar HSP90 (Krishna and Gloor, 2001). Queitsch et al. (2012) reported that the application of geldanamycin, a specific inhibitor of HSP90, to *Arabidopsis* plants elevated homologous recombination (HR) frequencies, suggesting that the HSP90s are involved in genome maintenance in plants.

Human HSP90 interacts with HsPolη and HsRev1 and regulates the TLS activities (Sekimoto et al., 2010; Pozo et al., 2011). The frequency of UV-induced supF mutation in hPolη-proficient cells is elevated by applying 17-AAG, an HSP90 inhibitor, due to the inhibition of error-free bypass of UV damage (Sekimoto et al., 2010). Conversely, in HsPolη-deficient cells, 17-AAG treatment reduces mutation due to the inhibition of the REV1-dependent error-prone bypass (Pozo et al., 2011). In contrast with the results in mammals, treatment with geldanamycin reduces mutation frequencies in wild-type plants, which are AtPolη-proficient (Sakamoto et al., 2018). This suggests that HSP90 mainly regulates the error-prone TLS pathway, involving AtRev1, in *Arabidopsis*.

## Translesion Synthesis and Cell Cycle Checkpoint

In *Arabidopsis*, UVB or gamma irradiation induces programmed cell death of stem and progenitor (StPr) cells in the root meristem that depends on *ataxia-telangiectasia mutated* (*ATM*), *ataxia-telangiectasia and Rad3-related* (*ATR*), and *SUPPRESSOR OF GAMMA RESPONSE1* (Curtis and Hays, 2011; Furukawa et al., 2010). Curtis and Hays (2011) investigated the time course of cell death in UV-irradiated Polζ-deficient (*rev3*) and Polη-deficient (*polh*) roots as well as the roots of damage checkpoint kinase *atm* and *atr* mutants. They found that the cells in *polh* plants started dying at around 16 h after UV treatment, but the cells in *rev3* plant started to die at around 20 h. The time courses of cell death in *atr* and *rev3 atr* plants were similar to that in *rev3* plants, whereas the UV dose-dependency plots of *atr, rev3 atr*, and *rev3* fitted similar slopes. Thus, they hypothesized that there are two types of TLS in *Arabidopsis* StPr cells: rapid TLS involving Polη and slow TLS involving Polζ. No Polη or a failure of rapid TLS results in the accumulation of single-stranded DNA, which activates a damage checkpoint and Polζ bypasses the damage (slow TLS). If both Polη and Polζ are absent, or if Polη and ATR are absent, then the stalled replication fork collapses to produce DSB, and the ATM activates DSB repair pathways. A similar epistatic relationship between ATR and Polζ was observed in yeast; the Polζ-dependent mutation requires the yeast ATR homolog Mec1 (Pagès et al., 2009). Therefore, some, but not all, of the TLS activities appear to be controlled by checkpoint activation in both plants and microorganisms.

REV7, the regulatory subunit of Polζ, contains a HORMA (Hop1, Rev7, and MAD2) domain (Aravind and Koonin, 1998). Based on its homology to MAD2, the key component of the mitotic-spindle-assembly checkpoint, and Hop1, a meiotic-synaptonemal complex component, it has been speculated that REV7 acts as an adaptor for DNA repairs and the spindle assembly checkpoint (Aravind and Koonin, 1998). Human REV7 makes a homodimer, and REV7-MAD2 a heterodimer, *in vitro* (Murakumo et al., 2000). In the absence of REV7, human cells arrest in the G2/M-phase and display increased monoastral and abnormal spindles with misaligned chromosomes (Bhat et al., 2015). Crystal structure and NMR analyses showed that two copies of REV7 bind to the canonical REV7-binding motifs (RBMs) of REV3 (Rizzo et al., 2018). In plants, *Arabidopsis* REV7 makes a homodimer in both the nucleus and the cytosol (Sakamoto et al., 2018). However, there is only one repeat of RBM in AtREV3 sequences, which is similar to yeast REV3 (Tomida et al., 2015). Therefore, the conformation of active Polζ in plants could be different from that in mammals.

## Translesion Synthesis Dna Polymerase in the Repair of Double-Strand Breaks or Crosslink Damage

Substantial evidence points to the involvement of TLS polymerases in the DSB repair pathway. For example, chicken REV3(−/−) cells and *Arabidopsis rev3* plants are sensitive to ionizing radiation (Sakamoto et al., 2003; Sonoda et al., 2003). *Arabidopsis* Polλ-disruption plants are hypersensitive to ionizing radiation and bleomycin (Furukawa et al., 2016). In yeast, Polζ and Rev1 are associated with the homing endonuclease (HO)-induced DSB end (Hirano and Sugimoto, 2006). Moreover, ScREV3 is responsible for mutations near the HO-induced cleavage site (Holbeck and Strathern, 1997; Rattray et al., 2002). These results show that some TLS polymerases, at least, have a role in DSB-repair processes in both animals and plants. Recently, DNA polymerase θ was shown to be involved in the alternative end-joining (Alt-EJ) pathway in animals (Chan et al., 2010; Wood and Doublié, 2016; Schimmel et al., 2017) and in moss (Mara et al., 2019). Polθ-deficient *Arabidopsis* cannot integrate T-DNA, suggesting that the Polθ stabilizes two minimally paired 3’ overhanging DNA ends during the T-DNA integration process (van Kregten et al., 2016). It is possible that TLS polymerases work in DSB repair pathway in some context.

Polθ is the best understood polymerase involved in the repair of interstrand crosslink (ICL) damage (Harris et al., 1999; Shima et al., 2003; Beagan et al., 2017). Other TLS polymerases have also been suggested to work in the process of ICL damage repair. For example, REV3(−/−) cells or organisms are sensitive to ICL-inducing treatments in mammals, chickens, yeast, and plants (Grossmann et al., 2000; Sakamoto et al., 2003; Nojima et al., 2005; Takahashi et al., 2005; Sarkar et al., 2006; Sharma et al., 2012). Disruption of Rev1 and Polη makes the cell or organism hypersensitive to ICL treatment (Takahashi et al., 2005; Sharma et al., 2012), and hPolη can bypass the ICL adduct *in vitro* (Vaisman et al., 2000). It has been suggested that ICL damage is processed by the Fanconi anemia complementation group A (FANCA)-dependent pathway, which includes nucleolytic incision, TLS, and HR (Kim and D’Andrea, 2012). Several TLS polymerases have been shown to bypass ICL damage if the DNA around the ICL is appropriately trimmed (Ho et al., 2011; Roy et al., 2016). These data suggest that TLS activities are important in overcoming ICL damage. In conclusion, TLS polymerases have multiple roles, which are critical for the genome stability of animals, plants, and microorganisms.

## Conclusion and Future Perspectives

The deletion of TLS polymerase often causes lethal or severe phenotypes in animals (Esposito et al., 2000; O-Wang et al., 2002; Wittschieben et al., 2006; Dumstorf et al., 2006; Stallons and McGregor, 2010). By contrast, almost all TLS polymerase activities can be disrupted in plants without severe reduction of fertility. Therefore, the plant system is ideal for analyzing the function, regulation, and interaction of TLS polymerases. Information on the structure and catalytic fidelity of TLS polymerases can assist us to build a novel genome-editing enzyme with elaborate specificities. Plant cells can provide a good platform for developing these upcoming technologies.

## Author Contributions

ANS wrote all the parts of this mini-review.

## Funding

This work was partially supported by Japan Society for the Promotion of Science (JSPS) KAKENHI Grant number 24241028, 25440147, and 17K00561 to AS.

## Conflict of Interest

The author declares that the research was conducted in the absence of any commercial or financial relationships that could be construed as a potential conflict of interest.
